# A Comparison Between Multisonic and Ultrasonic Irrigant Activation Techniques for Multispecies Biofilm Removal During Root Canal Disinfection: A Systematic Review

**DOI:** 10.7759/cureus.80938

**Published:** 2025-03-21

**Authors:** Preethi Varadan, Sangavi Ra, Mathan R Rajendran

**Affiliations:** 1 Conservative Dentistry and Endodontics, Sri Ramachandra Institute of Higher Education and Research Deemed-to-be-University, Chennai, IND

**Keywords:** activated irrigation, endodontic bacteria, real time pcr, root canal disinfection, ultrasonic irrigation

## Abstract

This systematic review aims to compare multisonic and ultrasonic irrigant activation techniques in polymicrobial biofilm removal. We conducted a literature search involving SCOPUS, PubMed, Cochrane, EBSCO host, and LILACS databases from inception to December 2024, in addition to a manual search using Google Scholar. The risk of bias assessment was done using the guidelines described for in vitro studies and the criteria for evaluation was based on JBI criteria and CRIS guidelines for quality assessment of in vitro studies. Three articles were included in the final analysis. The included studies evaluated biofilm removal in extracted teeth models incubated with biofilm; while two studies used quantitative polymerase chain reaction (qPCR) methodology, one used colony forming unit (CFU). Except in the study by Llerena et al., the GentleWave® multisonic System (GWS) outperformed ultrasonic irrigant activation (UI) in terms of biofilm removal. Despite the limitations of this systematic review, multisonic irrigant activation demonstrated similar efficacy in reducing intraradicular biofilm when compared to ultrasonic irrigant activation.

## Introduction and background

Endodontic infections are caused by different bacterial species organized in a mixed biofilm community, similar to most human endogenous infections, dental caries, and marginal periodontitis [1]. The canal irregularities such as apical ramifications, lateral canals, isthmuses, dentinal tubules, and recesses harbor bacteria, often organized in biofilm-like structures [2]. Planktonic cells and flocs in the main root canal are easily accessed and eliminated by instrumentation and irrigants. However, the biofilm adherent in the inaccessible areas distant from the main canal is challenging to remove. Biofilms persist due to their various mechanisms for evading treatment such as protection by the enclosing polysaccharide matrix, intrinsic resistance to endodontic disinfectants, and ability to survive and adapt to new environments by activating survival genes and following alternate metabolic pathways. Partial elimination of such endodontic microbes might result in the reorganization of species, predisposing to unfavorable treatment outcomes and flare-ups [1].

Root canal rendered free of biofilm is favored for obturation. However bacterial load reduction conducive to healing is the primary goal. From a microbiological standpoint, eradication or significant disruption of biofilm architecture is difficult. This is further challenged by the anatomical complexities of root canals which are impossible to sterilize [2]. Mechanical disruption of the tenacious biofilm structure and smear layer is required for broad-spectrum chemical disinfectants to exert their action on cells inside the matrix. Strategic delivery of irrigants to the canals is needed to potentiate the chemical effects of disinfectants [2].

Ultrasonic activation acts mainly by the agitation of the surrounding irrigant rather than a direct physical effect confined to the main root canal. At 30 kHz, the oscillatory motion of the tip generates acoustic streaming, which transports the irrigant from the main root canal to the remote zones. It increases the wall shear stress to improve the mechanical effect. Under certain circumstances, the swiftly changing irrigant pressure might foster transient acoustic cavitation. The higher shear wall stress along with locally increased pressure and temperature evoke sonochemical effects by accelerating the chemical reactions. Two types of ultrasonic irrigation have been elucidated in the literature: continuous ultrasonic irrigation (CUI) and passive ultrasonic irrigation (PUI) [3].

The mechanical efficacy of ultrasonic irrigant activation in biofilm removal from lateral morphological features in root canals and decontaminating dentinal tubules has been studied in monospecies, dual-species, and multispecies biofilm [4]. However, several studies have focused on bacterial load reduction in Enterococcus faecalis (E. faecalis) biofilm models, which is not representative of the antimicrobial efficacy in vivo [5]. GentleWave® System (GWS), a new irrigant activation system combining multisonic frequencies and negative apical pressure has been introduced for root canal disinfection with minimal preparation sizes. This multisonic system creates a broad range of frequencies that traverse the entire root through the irrigant. These broad-spectrum acoustic waves are believed to impart root canal cleanliness during the collapse of hydrodynamic cavitation bubbles [5]. Studies have demonstrated favorable results for GWS in removing biofilm, intracanal bacterial DNA, calcium hydroxide medication, residual debris, separated instruments, gutta-percha/sealer in retreatment, and calcifications [6].

The key difference between the two systems is that ultrasonic activation is performed in each canal separately, whereas GWS actively circulates the irrigant simultaneously in all root canals, thereby minimizing the working time. Ultrasonics solely depend on the acoustic streaming of an oscillating file whose effect is limited when used in narrow canals. However, with regard to multisonic systems such as GWS, irrigant activation is achieved by the production of a broad spectrum of sound waves that deliver irrigants throughout the root canal [6,7].

The decontamination of infected root canals is a crucial aspect of endodontic treatment, with effective antimicrobial strategies being essential for preventing persistent infections. Previous studies have compared the effectiveness of multisonic and ultrasonic activation techniques in reducing polymicrobial load. Some studies have reported the superiority of multisonics over ultrasonics, while others have demonstrated no significant difference in the reduction of microbial contamination. However, there remains a lack of consensus on the most effective technique for biofilm disruption and decontamination. Additionally, there are no comprehensive systematic reviews in the literature comparing the antibiofilm efficacy of these techniques when used with different endodontic irrigants. This systematic review aims to compare the antibiofilm efficacy of different ultrasonic and multisonic activations of endodontic irrigants in multispecies biofilms.

## Review

Research proposal and research question

The research proposal was registered in the PROSPERO database with the PROSPERO ID: CRD42024583277. The review question per Preferred Reporting Items for Systematic Reviews and Meta-Analyses (PRISMA) guidelines was as follows: “Does multisonic irrigant activation effectively remove intraradicular biofilm when compared to ultrasonic irrigant activation?”

Materials and methods

PICO(S) Analysis

Population (P): Polymicrobial biofilm models

Intervention (I): Multisonic irrigant activation

Comparison (C): Ultrasonic irrigant activation

Outcome (O): Biofilm removal Study design

Study design (S): In vitro studies

Eligibility Criteria

In vitro studies comparing ultrasonic and multisonic irrigant activation techniques performed exclusively on polymicrobial biofilm models were included in this systematic review. Studies with no available full text, review articles, conference abstracts, case reports, and studies evaluating monospecies biofilm removal were excluded.

Study Selection and Data Extraction

**Figure 1:**
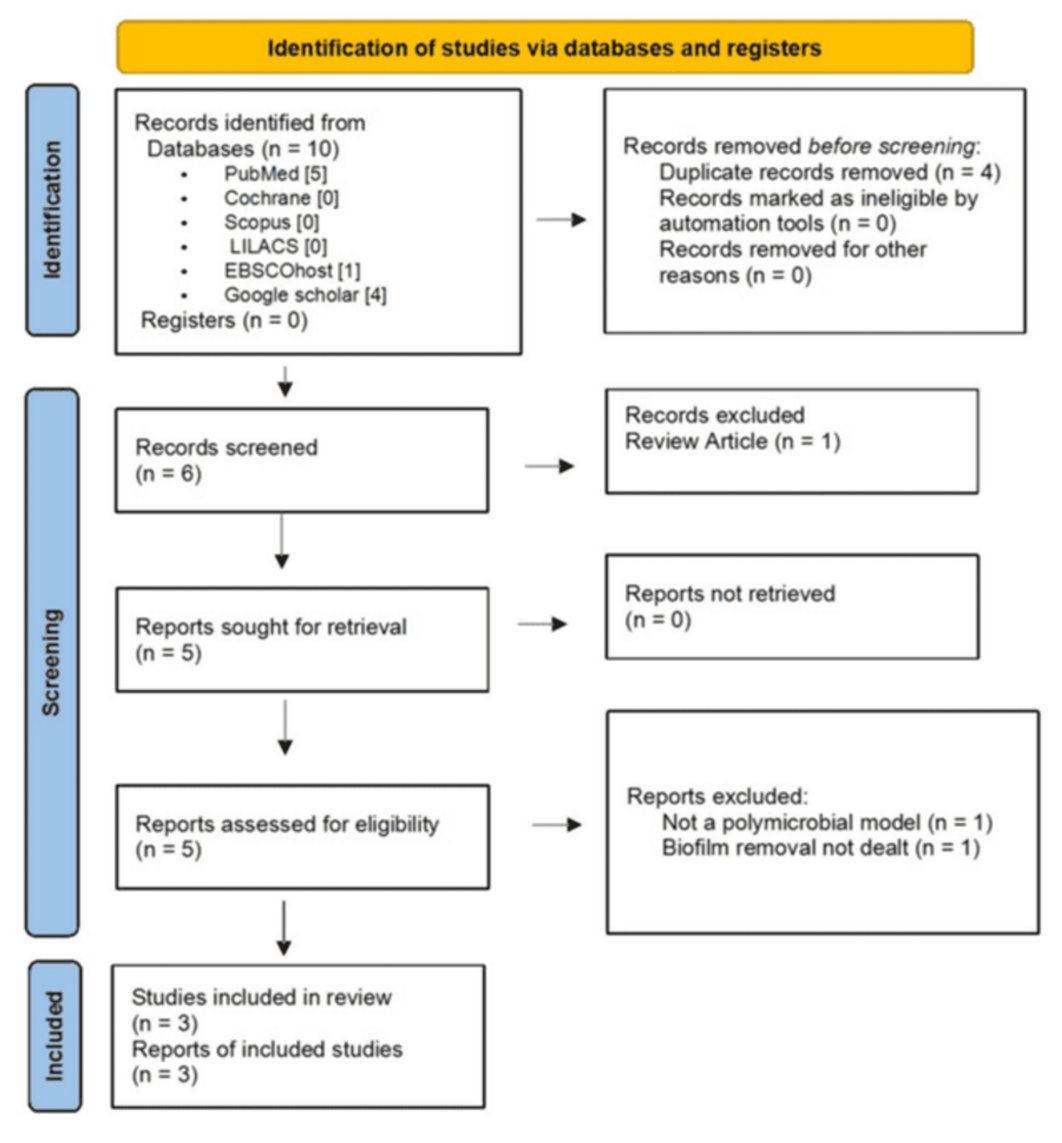
PRISMA flowchart illustrating the study selection process PRISMA: Preferred Reporting Items for Systematic Reviews and Meta-Analyses

A literature search was undertaken involving the databases PubMed, Cochrane, LILACS, SCOPUS, and EBSCO host from inception to December 2024, and a manual search was performed using Google Scholar from inception to December 2024. Two independent reviewers (PV and SR) screened the titles and abstracts of articles obtained from the preliminary search. Complete texts of the relevant studies were evaluated based on the inclusion criteria. A PRISMA flowchart detailing the study selection is depicted in Figure 1. The data extracted from the finalized articles included author name, year of publication, tooth type, sealing of apex, species tested, culture medium, incubation period, comparison groups, sample size, NiTi instrument used, apical preparation size (diameter and taper), irrigation protocol (duration, time, volume of irrigant), sample collection method, evaluation method for biofilm removal, results obtained and conclusion of the studies. Any disagreement in study selection was resolved by consulting with a third reviewer (MR).

Search Terms

The studies were selected with the use of search terms such as “Biofilm removal” OR “Biofilm eradication” OR “Biofilm disruption” OR “Biofilm detachment” OR “Biofilm disassembly” OR “Microbial reduction” OR “Multispecies biofilm” OR “Polymicrobial biofilm” OR “Dual species biofilm” OR “Polymicrobial biofilm models” “Ultrasonically activated irrigation” OR “Ultrasonic activation” OR “Passive ultrasonic irrigation” OR “CK file” OR “IrriSafe” OR “Endosonic Blue” “Multisonic irrigant activation” OR “Gentlewave system” OR “Cleanflow technology”.

Qualitative Analysis of the Included Studies

Three independent analysts evaluated the methodological quality of the studies using the guidelines reported for in vitro studies. The evaluation criteria adopted were modified from the CRIS and JBI guidelines for quality assessment of in vitro studies. In case of discrepancies between the two examiners, a third reviewer was consulted to sort out the disagreement. The risk of bias assessment criteria included sample size estimation, sample preparation and handling, randomization of samples, biofilm standardization, incubation period, presence of a control group, and appropriate statistical analysis.

Results

Ten articles were identified from the preliminary search. After removing duplicates, the screening of titles and abstracts was performed for five studies, after which one article was excluded as it did not meet the inclusion criteria. The excluded studies and the reason for their exclusion are summarized in Table 1. On full-text screening of the remaining four articles, one article was excluded as it did not evaluate biofilm removal. Finally, three articles [5,7,8] were included for qualitative analysis. The clinical characteristics of the included studies are summarized in Tables 2-3.

**Table 1:** List of excluded articles

S.no	Author and year of publication	Reason for exclusion
1	Choi et al., 2019 [9]	The study used monospecies biofilm model
2	Velardi et al., 2022 [6]	The study did not evaluate biofilm removal

**Table 2:** Characteristics of the included studies BHI: brain heart infusion; GWS: GentleWave® System; PUI: passive ultrasonic irrigation

Study	Type of teeth	Sealing of apex	Species tested	Culture medium	Incubation period	Groups	Apical preparation size	Results	Conclusions
Coaguila-Llerena et al., 2022 [5]	22 lower molars (Vertucci type II)	Sealed	10 bacterial taxa, including Streptococcus, Parvimonas, Fusobacterium, Prevotella, Veillonella, Mogibacterium, Slackia, Selenomonas, Stomatobaculum, and Lancefieldella, were tested	BHI broth	2 weeks	Group GWS (n = 11); Group PUI (n = 11)	For GWS: V-Taper 20.06. For ultrasonic: Vortex Blue 35.04	Both groups had no differences in results between pre- and post-treatment samples. Pre-treatment communities differed from post-treatment samples in both groups regarding bacterial taxa reduction	GWS and PUI showed a similar bacterial reduction
Zhang et al., 2018 [7]	20 extracted human molars	Sealed	E. faecalis and Streptococcus mutans were tested	BHI broth	2 weeks	Group GWS (n = 26 canals); Group PiezoFlow (n = 30 canals)	For GWS: Vortex Blue 15.04. For PiezoFlow: Vortex Blue 35.04	The reduction in the mean number of bacteria was 99.91% for GWS and 98.01% for the PiezoFlow system	GWS showed a high level of bacterial DNA reduction in comparison to the PiezoFlow system
Alquria et al., 2023 [8]	70 single-rooted lower premolars (Vertucci type I)	Not reported	E. faecalis and E. coli were investigated	BHI broth	3 weeks	1) Positive control group (n = 14); 2) GWS + minimally invasive technique group (MIT) (n = 14); 3) PUI (ProUltra) + conventional instrumentation technique group (n = 14); 4) XP-Endo Finisher (XPEF) + conventional instrumentation technique group (n = 14)	For GWS: Vortex Blue 20.04. For PUI: Vortex Blue 35.04	PUI + conventional instrumentation technique, XPEF + conventional instrumentation technique, and GWS + minimally invasive technique were highly effective against bacteria with GWS with MIT being the most effective	GWS + minimally invasive technique was the most effective

**Table 3:** Irrigation protocols in the included studies GWS: GentleWave® System; PUI: passive ultrasonic irrigation

Study	Irrigation protocol	Volume of irrigants	Methods of sampling	Evaluation methods
Coaguila-Llerena et al., 2022 [5]	For PUI, a 20.02 size ultrasonic tip was coupled to a piezoelectric device (EndoUltra), and 2 ml of the solution was activated for 20 secs. The procedure was repeated thrice for a total final irrigation time of 1 minute. The same procedure was repeated for 17% ethylenediaminetetraacetic acid (EDTA). GWS was used at a 1-min cycle with distilled water, a 4-min cycle with 3% sodium hypochlorite (NaOCl), a 1-min cycle with 8.5% EDTA, and a 1-min cycle with distilled water finally. After final irrigation, all the canals were irrigated with 2 ml of 10% sodium thiosulfate (STS) for 3 min to inactivate the NaOCl	For PUI, the total NaOCl volume of final irrigation per canal was 6 ml. However, for GWS, the volume was not reported	Paper point sampling method	Quantitative polymerase chain reaction (qPCR), 16S ribosomal RNA gene sequencing (next-generation sequencing) method was used
Zhang et al., 2018 [7]	For ProUltra PiezoFlow, the irrigation protocol used was 3% NaOCl for 1 min per canal, sterile water for 10 s per canal, 8% EDTA for 1 min per canal, and sterile water for 10 s per canal. For GWS, the irrigation protocol used was 3% NaOCl for 3 mins, sterile water for 30 s, 8% EDTA for 2 min, and final irrigation with sterile water for 15 secs	Not reported	Paper point sampling method	Both the 16S ribosomal RNA gene-targeted qPCR method and culturing method were used
Alquria et al., 2023 [8]	For PUI, 5 ml of 3% NaOCl for 3 mins followed by 5 ml of 17% EDTA for 3 mins, and a final flush of 5 ml of 3% NaOCl for 3 mins was used. To inactivate NaOCl, 5 mL sterile 0.5% STS for 1 min was used. For GWS, an irrigation protocol of 3% NaOCl for 5 min, sterile water for 15 s, 17% EDTA for 2 min, and a final flush of sterile water for 15 s was used. The root canals were finally irrigated with 5 mL sterile 0.5% STS for 1 min and rinsed with sterile saline solution (SSL) to inactivate NaOCl. For XP-Endo Finisher, the irrigation protocol was 17% EDTA for 1 min and 5 mL of 3% NaOCl. This protocol was repeated 2 times. A final flush of 3% NaOCl was performed and the NaOCl effect was inactivated with 5 mL sterile 0.5% STS for 1 min	Not reported	Paper point sampling method	Colony forming unit (CFU) method was used

All the studies were published between 2018 to 2023. Among the included studies, two used molars, while Alquria et al. [8] utilized premolars for biofilm incubation. Regarding the biofilm models, two studies sealed the apex of the tooth, whereas Alquria et al. [8] did not mention whether the apex was sealed in their study. Zhang et al. [7] grew three-week-old E. faecalis and Streptococcus biofilms while Alquria et al. [8] used two-week-old E. faecalis and E. coli dual-species biofilms in their study. Llerena et al.'s study [5] study predominantly focused on 10 bacterial taxa (Streptococcus, Parvimonas, Veillonella, Mogibacterium, Fusobacterium, Prevotella, Slackia, Selenomonas, Stomatobaculum, and Lancefieldella) for two weeks. All the studies used BHI broth, which is an anaerobic culture medium. Except in the study by Alquria et al. [8], a control group was not included, and randomization of samples was not performed.

For both CUI & PUI, all the studies used Vortex blue NiTi instruments till 35.04 for canal preparation. For GWS, the apical preparation size was different for each study. Llerena et al. [5] had used V-taper till 20.06. Vortex blue had been used till 35.04 by Alquria et al. [8] and 15.04 by Zhang et al. [7]. None of the studies mentioned the final irrigant volume used for each group [5,7,8]. After final irrigation, all canals were irrigated with sodium thiosulfate to inactive the sodium hypochlorite (NaOCl) carry-over effect, except Zhang et al. [7]. All the studies had collected samples from the region of interest using paper points.

All of the included studies evaluated biofilm removal in extracted teeth models incubated with biofilm; two studies used quantitative polymerase chain reaction (qPCR) methodology, and the other study used colony forming units (CFU). In all studies except Llerena et al. [5], GWS outperformed PUI in biofilm removal.

Risk of Bias Assessment

The evaluation criteria were revised based on the CRIS and JBI guidelines for assessing the quality of in vitro studies. Two of the three included studies displayed a high risk of bias while one showed a moderate risk of bias. None of the authors had revealed the sample size derivation. Tooth randomization was done in Alquria et al.'s [8] study. All the studies had specified details about sample preparation and handling, biofilm standardization, incubation period and statistical analysis performed. Alquria et al. [8] had a control group while the remaining studies had no control group. Regarding irrigation protocols in treatment groups, Alquria et al. [8] did not mention the working length till the ultrasonic tip was inserted and the frequency with which PUI was operated. In Llerena et al. (2022) [5] and Zhang et al. (2019) [7], the power setting of PUI is not specified. The quality assessment of the included articles is presented in Table 4.

**Table 4:** Quality assessment of included studies The studies were categorized as a “high risk of bias” (score: 0–3), “moderate risk of bias” (score: 4–6), and “low risk of bias” (score: 7-8) +: Yes (if the defined question was answered in the article). -: No (if the defined question was not answered in the article). ?: Unclear (if the defined question was clearly given in the article)

Author	Sample size calculation	Tooth randomization	Sample preparation and handling	Biofilm standardization	Incubation period	Control group	Appropriate statistical analysis	Total score
Coaguila-Llerena et al., 2022 [5]	-	-	+	+	+	-	+	4
Duo et al., 2018 [7]	-	-	+	+	+	-	+	4
Alquria et al., 2023 [8]	-	+	+	+	+	+	+	6

Discussion

None of the selected studies included a priori sample size calculation in the study design. Inadequate sample size and methodological flaws in sampling and detection lead to erroneous inferences about different automated irrigant activation techniques. This mandates researchers to effectuate changes in in vitro models to study the root canals relevant to the clinical scenario. Culture methods allow either an absolute or partial quantification of culturable bacteria. However, under laboratory conditions, a significant number of microbiotas in the root canal space cannot be cultured. E. faecalis appeared to be commonly associated with persistent or secondary endodontic infections based on culture and closed-end methods such as PCR during the last two decades [8-12]. This has been challenged by contemporary studies that shed light on a complex bacterial composition evident in failed cases. Mature biofilm differs from an early biofilm with respect to biomass and antimicrobial resistance. All the included studies in this review designed mature biofilm models.

Gram-negative species are uncommon in post-instrumentation or medication samples, which indicates that they are easily eliminated, unlike gram-positive bacteria. Since gram-positive species are the most challenging to eliminate, their usage is justifiable. Because a given treatment might have different effects on gram-negative and gram-positive bacteria, studies have included both species [13]. Llerena et al. [5] used dental plaque samples, which are undefined natural biofilms, as well as a CDC reactor to generate reproducible microcosm biofilms [14]. Zhang et al.'s [7] study used a multispecies biofilm comprising E. faecalis and Streptococcus mutans. Alquria et al. [8] used a dual-species biofilm model comprising E. coli LPS, which is commercially available and accessible. However, this gram-negative species is not usually found in root canal infections.

All the included studies employed sterile paper points for sample collection. Although paper point samples are routinely used for intracanal microbiological studies, they do not reach inaccessible regions like accessory canals, fins, and dentinal tubules where residual biofilms persist. Underestimation of the intraradicular infection levels and biofilm removal from those inaccessible regions is likely to result from this technique. To overcome that limitation, cryogenic grinding was used in Alquria et al. [8]. However, if the tooth is pulverized, sequential sampling cannot be done.

Desired features of ideal irrigation methods include the ability to deliver the irrigant to the complete root canal system, refresh the irrigants to compensate for consumption, and apply shear stress on targets to detach them from the canal wall, thus manifesting a reverse flow to carry detached materials and depleted irrigant out of the canals and prevent inadvertent extrusion. Although irrigation protocol and biofilm model were standardized in terms of canal anatomy and microbiology, it is impractical to match the irrigant volume, canal preparation diameter, and taper, as different numbers of files were used in each group.

PUI should be carried out with an apical preparation size of 30-35 for unobstructed oscillation. A higher power setting leads to more intense streaming, but the dentist should be mindful of instrument separation and inadvertent dentin removal. Most manufacturers recommend 30-35 % of maximum available power. Shorter periods of intermittent activation combined with irrigant replenishment is commonly used than a high-flow rate continuous delivery system. Intermittent activation involving three cycles of 20 seconds is a popular protocol, though a shorter time of 10 seconds can also be employed. Continuous ultrasonic activation creates high shear wall stress at the apical third resulting in an improved reduction of adherent biofilm than intermittent ultrasonic irrigation and conventional syringe irrigation. However, the PUI power setting is not mentioned in the studies by Llerena et al. [5] and Zhang et al. [7].

Multisonic activation was promoted as a stand-alone irrigation method and the GW system is designed to work on minimal (apical enlargement to size 15-25) or even uninstrumented canals. Conversely, a study by Lee et al. (2019) [14] stated that apical diameter is not a relevant factor if irrigants are activated. Hence, the results with the minimal preparation sizes with GWS are favorable in preserving the pericervical dentin without compromising the disinfection, ultimately retaining the resistance against tooth fracture. Also, it produces negative apical pressure, which is believed to prevent irrigant extrusion significantly. To eliminate the dissolved gas present in the solution and eliminate the vapor lock effect, the irrigants undergo a degassing process. When the irrigant moves from the GW handpiece into stagnant fluids in the pulp chamber, the shear forces trigger hydrodynamic cavitation, and cavitation clouds are formed. The implosion of these microbubbles generates sound waves that reverberate via the root canal. The handpiece has a 5-point vented suction system to collect excess irrigant from the pulp chamber [12].

The recommended protocol involves 3% NaOCl irrigant activation for three or five minutes, subsequently irrigated with water for 15 or 30 seconds, 8% EDTA for two minutes, and finally distilled water rinse for 15 or 30 seconds [12]. In Alquria et al.'s study [8], 17% EDTA is used. The interplay of multisonic energy, vortical fluid dynamics, and irrigant chemistry is believed to enhance the removal of organic debris from the root canal system [12]. This could have possibly been attributed to the leverage in disinfection as seen in the studies of Zhang et al. [7] and Alquria et al. (2022) [8].

GWS handpiece dispenses irrigants at a high-speed shear force of about 45 ml/min flow rate. Thus 180 ml of NaOCl and a total volume of 270 ml irrigants are utilized. Matching this irrigant volume in an ultrasonic system by increasing the activation time increases the risk of ultrasonic tip separation, ledge, and uncontrolled dentin removal. Despite the different mechanisms of action, bacterial reduction was similar in both GWS and ultrasonic activation groups in the study by Llerena et al. [5].

From this review, it can be noted that ultrasonic irrigant activation is on par with GWS in biofilm removal and is feasible. At the same time, GWS is highly effective in simultaneous irrigant activation in all the canals, thereby reducing the chairside time for disinfection though it requires a minimum time to build a platform at the cavosurface before beginning the endodontic disinfection. More importantly, maintenance issues and monitoring the long disinfection cycle of the entire GW console do not surface when it comes to ultrasonic units. Thus, GWS requires a brief learning curve to endorse it into routine clinical practice [12]. Future in vivo studies can deal with the skepticism regarding postoperative pain and hemorrhagic episodes following multisonic activation.

This review provides a good starting point to advocate for a customized irrigant activation protocol based on the microbiome and anatomy of the root canal system in each patient. In conjunction with an overly simplified bacterial killing and biofilm elimination approach with different irrigants and activation methods, future research can focus on a multifaceted strategy deploying targeted antimicrobial peptides to disrupt the EPS matrix, suppress the virulence factors and stress-associated bacterial genes, and kill dormant and persistent cells.

Highlights

Multisonic irrigant activation displayed similar efficacy in biofilm removal when compared to ultrasonic irrigant activation. Volume and duration of irrigant usage are inherently different in both GWS and ultrasonic activation, which influences biofilm removal. GWS is highly effective in simultaneous irrigant activation in all the canals, thereby reducing the duration of disinfection.

## Conclusions

Multisonic irrigant activation demonstrated similar efficacy in reducing the biofilm when compared to ultrasonic irrigant activation. However, the multisonic system proved to be advantageous in reducing chairside time because of the simultaneous irrigant activation in all the canals. Future in vivo studies should focus on case-specific irrigation protocol by incorporating knowledge from chemistry, microbiology, and fluid dynamics.
